# Midlife physical activity, BMI, and hip fracture risk five decades later in men: a NOREPOS study

**DOI:** 10.1007/s11657-026-01657-1

**Published:** 2026-01-26

**Authors:** Ida Kalstad Landgraff, Marius Myrstad, Anette Hylen Ranhoff, Ove Talsnes, Kristin Holvik, Haakon E. Meyer

**Affiliations:** 1 Department of Internal Medicine, Bœrum Hospital, Vestre Viken Hospital Trust, Gjettum, Norway; 2 Faculty of Medicine, Institute of Clinical Medicine, University of Oslo, Oslo, Norway; 3 Department of Medical Research, Bœrum Hospital, Vestre Viken Hospital Trust, Gjettum, Norway; 4 Department of Internal Medicine, Diakonhjemmet Hospital, Oslo, Norway; 5 Department of Orthopedics, Innlandet Hospital Trust, Elverum, Norway; 6 Department of Chronic Diseases and Ageing, Norwegian Institute of Public Health, Oslo, Norway; 7 Department of Community Medicine and Global Health, University of Oslo, Oslo, Norway

**Keywords:** Physical activity, Exercise, Hip fracture, Population-based studies, Men, Fragility fractures, Epidemiology

## Abstract

**Summary:**

Research on hip fracture prevention in men is limited. In men, physical activity and body mass index were independently and jointly associated with hip fracture risk, with the highest risk among inactive and thin men. Promoting exercise and healthy weight in midlife may reduce fracture burden and support healthy ageing.

**Purpose:**

Hip fractures predominantly affect older people with frailty. The incidence increases with age, and the number is expected to increase substantially due to population aging. Physical inactivity and low body mass index (BMI) are key modifiable risk factors for hip fractures. This study aimed to explore the associations of physical activity and BMI with long-term hip fracture risk in men.

**Method:**

This prospective cohort study included 12,900 men aged 40**–**49 years from the Oslo study 1972**–**1973. A questionnaire assessed physical activity, whereas height and weight were measured. Hip fractures were identified through linkage to a national database. Cox regression calculated adjusted hazard ratios (HR) with 95% confidence intervals (CI) for hip fracture according to physical activity level. A secondary analysis examined a composite variable combining activity level (inactive/active) and BMI (< 22/22–24.9/ ≥ 25 kg/m^2^).

**Results:**

During 195,384 person-years of follow-up, 1542 men (12%) sustained a hip fracture at a median age of 82 years. Inactive men had a 38% higher risk (HR 1.38 [95% CI 1.16**–**1.63]) than active men. Active men had a lower hip fracture risk across all categories of BMI, while the greatest risk was found in inactive men with a BMI < 22 kg/m^2^, HR 1.79 [95% CI 1.36**–**2.35] compared to active men with a BMI ≥ 25.

**Conclusions:**

Physical inactivity and low BMI in midlife were independently associated with increased long-term hip fracture risk. Inactive and thin men had the greatest risk, suggesting that maintaining physical activity in mid-life is important for healthy ageing and independence.

## Introduction

Hip fractures are a major health concern, particularly among older adults living with frailty. These fractures are usually a result of low-energy trauma and are associated with significant functional decline, loss of independence, and excess mortality. These consequences profoundly impact quality of life, with men experiencing a notably higher excess mortality rate following a hip fracture compared to women [1–3].

The hip fracture incidence increases with age [4]. At age 50, the lifetime risk of a hip fracture in men can be as high as 11% [5], making it a significant concern for the older population. Despite a decline in the hip fracture incidence observed over the past decades [6, 7], the growing population aged 65 years and older will lead to a numerical increase. A recent report on secular trends in incidence rates showed that the total number of hip fractures is projected to nearly double by 2050, suggesting that hip fractures are likely to remain a major challenge for the older population and the health care system [8]. Notably, the report predicted a larger increase in the hip fracture incidence in men than women. Given these trends, it is important, at a population level, to strengthen preventative efforts throughout the lifespan.

Physical activity is a modifiable risk factor for hip fractures, as it mitigates age-related bone loss, while enhancing muscle strength, balance, and coordination [9]. These factors are imperative for reducing the risk of falls and fractures in older people. However, the level of physical activity in the general population does not meet the recommended amount [10]. A more sedentary lifestyle has been adopted [11], with increased risk of developing conditions associated with reduced physical activity [12, 13]. Furthermore, the level of physical activity in different phases of life may be associated differently with long-term hip fracture risk.

Body mass index (BMI) is another important modifiable risk factor, playing a distinct but interrelated role with physical activity [14]. Previous research has shown an inverse relationship between BMI and hip fracture risk [15, 16]. A higher BMI stimulates bone formation through mechanical loading and an increased strain on the weight-bearing skeleton [17, 18]. However, the protective association is strongest at moderate values of BMI and seems to plateau around a BMI of 25 kg/m^2^, with limited benefit above this threshold [19, 20].

Understanding the interplay between physical activity level and BMI, and their association with hip fracture risk could be of importance in reducing hip fracture incidence, preventing loss of independence, and improving quality of life in older people. While the relationship between physical activity levels, BMI, and hip fracture risk has been extensively studied in postmenopausal women [21, 22], the evidence in men remains limited. This study aimed to examine the association between midlife leisure-time physical activity level and BMI in men, and the hip fracture risk up to five decades later.

## Materials and methods

### Design and study population

In this prospective cohort study, data from a population-based health study among men residing in the Norwegian capital of Oslo in 1972**–**1973 were linked to a national hip fracture database.

#### The Oslo study 1972–1973

All men residing in Oslo in 1972**–**1973 and born during the years 1923**–**1932 (age 40**–**49) were invited to participate in a health study screening for cardiovascular disease and its risk factors as the primary purpose. Participation included a questionnaire and a simple physical examination. The questionnaire, which the participants were instructed to fill out at home, provided information about medical history, lifestyle, and health behavior. A total of 16,205 men aged 40–49 years participated. Of these, 2908 (17.9%) died and 49 (0.3%) emigrated before 1994, leaving 13,248 men alive and residing in Norway by 1 January 1994 (see below). The study population eligible for analysis included those who had responded to the question about leisure-time physical activity level and had valid data on height, weight, and smoking, comprising 12,900 men. The design of the Oslo study has been described previously [23, 24].

#### Hip fractures

The NOREPOS hip fracture database, NORHip, provided information on all hip fractures treated in Norwegian hospitals from 1 January 1994 (which is the first year all Norwegian hospitals used electronic patient administrative systems) until 31 December 2018. Data from hospital admissions with a hip fracture diagnosis code (ICD-10: S72.0, S72.1, S72.2) was collected from patient administrative systems of treating hospitals for the period 1994 to 2007 and from the Norwegian Patient Registry from 2008 and onwards. The NORHip database is close to complete as virtually all patients who suffer a hip fracture in Norway are surgically managed in hospital. A validated comprehensive algorithm (www.norepos.no/documentation) was used to prepare raw data, incorporating details from each hospital admission such as co-occurring diagnosis codes, surgical procedure codes, and whether the hip fracture was a primary or secondary diagnosis to identify newly arisen (incident) hip fractures. In this analysis, only the first hip fracture occurring after the age of 62 years in each participant was included.

### Measurements

#### Self-reported physical activity

Information on self-reported physical activity was collected through a questionnaire filled out at home and included the validated Saltin-Grimby Physical Activity Level Scale [25, 26]: “State your bodily movement and physical exertion in leisure time. If your activity varies widely, for example between summer and winter, then give an average. The question refers only to the last twelve months.” The following four answers were mutually exclusive:

- 1: Reading, watching TV, or other sedentary activities.
- 2: Walking, cycling, or other forms of exercise at least four hours per week.
- 3: Participation in recreational sports, heavy gardening etc. at least four hours per week (including walking or cycling to work, Sunday walking, etc.).
- 4: Participation in hard training or sports competitions regularly, several times a week.

Category one and two will hereafter be referred to as “inactive” and “moderate,” respectively. Categories three and four were combined due to the small number of participants in the latter and are collectively referred to as “high” physical activity.

#### Other measurements

BMI was defined as weight in kilograms per height in meters squared and calculated from height and weight measured at the physical examination. The measurements were performed according to a standard protocol. Other relevant variables included self-reported information on cardiovascular disease, diabetes, and smoking. We retrieved data on level of education from Statistics Norway.

### Statistical analyses

Statistical analyses were performed using Stata version 18.0 (Stata Corp., College Station, TX). We used Pearson’s chi-square test of independence for categorical variables to compare characteristics between categories of leisure-time physical activity level, and Student’s *t*-test for continuous variables. A *p*-value of less than 0.05 was considered statistically significant. Categorical variables are reported as proportions (%) and continuous variables as means ± standard deviation (SD). The incidence rate was calculated as number of hip fracture cases divided by person-time at risk and reported as the number of hip fractures per 1000 person-years. Each participant who was alive and resided in Norway January 1, 1994 (first available date of hip fracture) was followed onwards to the date of hospital admission for the first incident hip fracture, emigration, death, or December 31, 2018, whichever came first. Information about dates of emigration and death was obtained from Statistics Norway. We excluded participants with missing data on height (0.7%), weight (0.7%), or education (0.2%), as well as participants with known cardiovascular disease (1.6%) or diabetes (0.4%) at participation in the Oslo study.

We performed Cox proportional hazards regression using attained age as timescale to estimate hazard ratios (HR) with 95% confidence intervals (CI) for hip fractures according to leisure-time physical activity level, with the highest physical activity level as reference group. Two models were constructed, one unadjusted (accounting for age only) and one adjusted for height, smoking, and level of education. BMI may act both as a mediator and a confounder in the association between physical activity and hip fracture risk. As a confounder, BMI may influence both physical activity level and hip fracture risk through differences in body composition and bone strength. Considering BMI as a mediator, part of the effect of physical activity on fracture risk may be caused through changes in body weight and composition. Higher levels of physical activity may lead to weight reduction, which in turn could result in a higher fracture susceptibility. At the same time, physical activity enhances bone strength, muscle mass, balance, and coordination decreasing fracture risk. As we considered BMI a mediator rather than a confounder, we did not include BMI as covariate. Furthermore, we carried out a flexible parametric survival analysis [26] with four degrees of freedom to allow HRs to vary over attained age, to visually assess how the risk of hip fracture across increasing age differed by midlife physical activity level. In this analysis, inactive men (the lowest response category) were compared to active men (the three highest response categories combined).

A statistical analysis of interaction between BMI as a continuous variable and leisure-time physical activity level was assessed by the log likelihood test. We conducted additional analysis to explore whether the influence of leisure-time physical activity on fracture risk varied across levels of BMI. For this analysis, we created a composite exposure variable with six levels, combining physical activity level (dichotomized into inactive and active (consisting of moderate and high level of physical activity)) and BMI (categorized as < 22 kg/m^2^, ≥ 22 to < 25 kg/m^2^, and ≥ 25 kg/m^2^).

Finally, we performed two sensitivity analyses where participants were censored when reaching age 85 and 80 years, respectively.

## Results

Out of the 13,248 participants in the Oslo study 1972**–**1973, 12,900 (97.4%) were included in the analysis. Among the included participants, 20% were physically inactive, 60% had a moderate level of leisure-time physical activity, and 20% reported the highest level of leisure-time physical activity (Table 1). Mean age at baseline was 44.6 years (SD 2.8). The group of inactive men had a slightly higher body weight, a higher BMI, were more likely to be smokers, and had lower levels of education compared with the active groups (*p* < 0.05 for all differences).

**Table 1:** Baseline characteristics across levels of midlife physical activity in men. The Oslo study 1972–1973

	Physical activity level		
	Inactive^a^	Moderate^b^	High^c^
	*n* = 2653 (20%)	*n* = 7892 (60%)	*n* = 2689 (20%)
Age (years) mean (standard deviation)	44.4 (2.9)	44.7 (2.9)	44.5 (2.6)
Height (cm) mean (standard deviation)	177.1 (6.7)	177.5 (6.4)	178.2 (6.1)
Weight (kg) mean (standard deviation)	78.8 (11.3)	77.5 (9.9)	77.4 (9.1)
BMI (kg/m^2^) mean (standard deviation)	25.1 (3.1)	24.6 (2.7)	24.4 (2.4)
Cigarettes per day, *n* (%)			
None	939 (42.3%)	3762 (55.0%)	1530 (64.9%)
1–14	715 (32.2%)	2010 (29.4%)	573 (24.3%)
15 or more	567 (25.5%)	1067 (15.6%)	255 (10.8%)
Education, *n* (%)			
Primary/lower secondary school	929 (36.1)	2046 (26.6)	563 (21.2)
Upper secondary school	1213 (47.0)	3794 (49.4)	1339 (50.6)
College/University/PhD	432 (16.9)	1838 (23.9)	746 (28.2)

^a^Inactive: Reading, watching TV, or other sedentary activity

^b^Moderate: Walking, cycling, or other forms of exercise at least 4 h per week

^c^High: Participation in recreational sports, heavy gardening etc. at least 4 h per week (including walking or cycling to work, Sunday walking) or participation in hard training or sports competitions regularly, several times a week

During a total of 195,384 person years of follow-up, 1542 men (12%) sustained a hip fracture. The mean follow-up time was 15.1 years from 1 January 1994. Median age at the time of the first hip fracture was 82 years. The incidence rate of hip fractures was 9.4 per 1000 person-years in the inactive group, compared to 7.1 per 1000 person-years in the highly active group, representing a risk difference of 2.3 per 1000 men (Table 2). We found an inverse association between leisure-time physical activity level and risk of hip fractures. In the adjusted model, inactive men had a 38% higher risk of hip fracture (HR 1.38 [95% CI 1.16**–**1.63]) compared to the most active men, whereas men in the moderately physically active group had a similar risk to the highly active men (Table 2).

**Table 2:** Hip fracture incidence rates (IR) and hazard ratios (HR) with 95% confidence intervals (95% CI) for hip fracture according to level of physical activity, using attained age as timescale

Physical activity level	*n*	Hip fracture events	Person-years	IR per 1000	Hazard ratio (95% CI)	
					Unadjusted	Adjusted^a^
High^b^	2648	301	42,380	7.1	1 (reference)	1 (reference)
Moderate^c^	7678	899	116,753	7.7	1.10 (0.96–1.25)	1.04 (0.90–1.20)
Inactive^d^	2574	342	36,251	9.4	1.49 (1.28–1.74)	1.38 (1.16–1.63)

^a^Adjusted for height, smoking, and level of education

^b^High: Participation in recreational sports, heavy gardening etc. at least 4 h per week (including walking or cycling to work, Sunday walking) or participation in hard training or sports competitions regularly, several times a week

^c^Moderate: Walking, cycling, or other forms of exercise at least 4 h per week

^d^Inactive: Reading, watching TV, or other sedentary activities

**Fig. 1:**
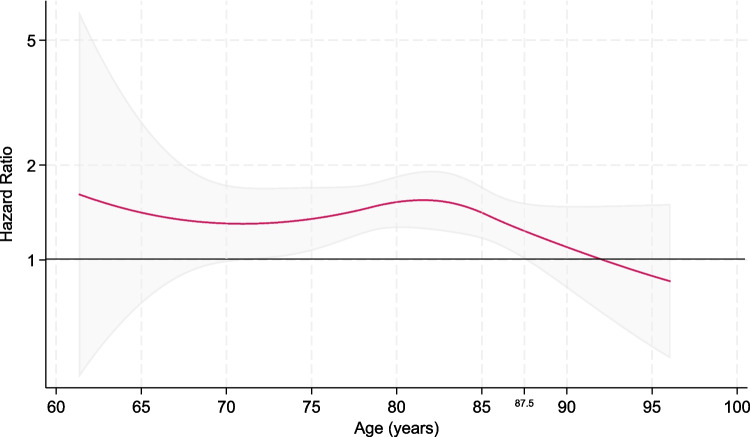
Hazard ratios, with 95% confidence intervals, for hip fracture across attained age in men who reported being physically inactive in leisure-time (n = 2648), compared with men who reported moderate or high physical activity levels (n = 10 252) in the Oslo Study 1972–1973. The hazard ratios are estimated in a flexible parametric survival model with three knots. The model is adjusted for height, smoking, and level of education

In a flexible parametric survival model, the HR of hip fracture in inactive men compared to all active men varied somewhat over attained age but was relatively stable up to approximately 85 years (Fig. 1). The increased hip fracture risk in inactive compared with active men was statistically significant in the age range 70**–**87.5 years.

**Fig. 2:**
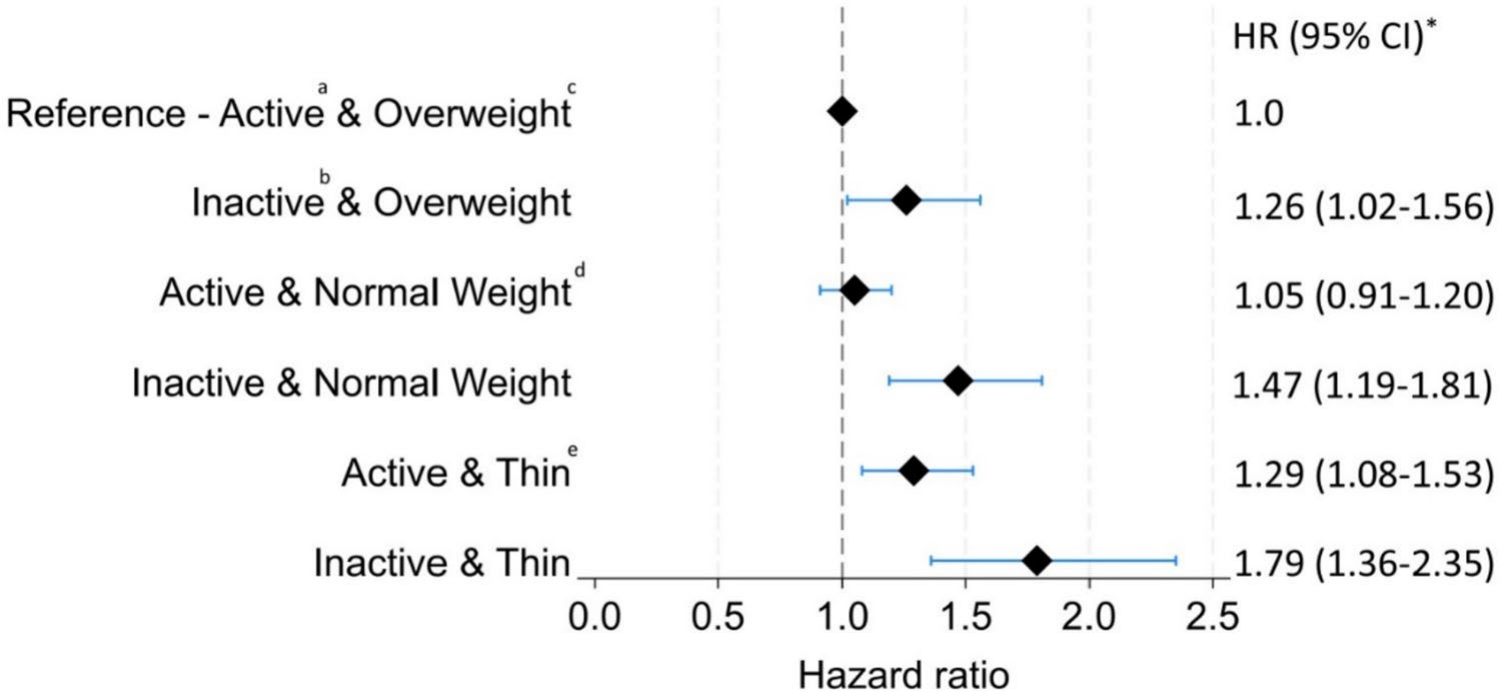
Forest plot showing hazard ratios with 95% confidence intervals for hip fracture events in six groups of men based on midlife physical activity and BMI. *Adjusted for height, smoking, and level of education. ^a^Active = self-reported light, moderate, or high levels of leisure-time physical activity. ^b^Inactive = no physical activity. ^c^Overweight = BMI ≥ 25 kg/m^2^. ^d^Normal weight = BMI ≥ 22 and < 25 kg/m^2^. ^e^Thin = BMI < 22 kg/m.^2^

The test for interaction between leisure-time physical activity level and BMI was not statistically significant (*p* = 0.47). When calculating HRs for physical activity and inactivity across three groups of BMI (< 22, 22–24.9, ≥ 25 kg/m^2^), men who were both inactive and had a BMI of less than 22 kg/m^2^ had the highest HR of hip fracture: 1.79 [95% CI 1.36**–**2.35] compared to active men (defined as a moderate or high physical activity level) with a BMI ≥ 25 kg/m^2^ (Fig. 2). Active men with a BMI of 22–25 kg/m^2^ had a comparable risk to those with a BMI ≥ 25 kg/m^2^ (HR of 1.05 [95% CI 0.91**–**1.20]).

In the sensitivity analyses where all men were censored at the age of 80 and 85 years, respectively, the results did not differ materially from those of the main analysis.

## Discussion

In this large prospective cohort study in men with hip fracture data available up to 47 years after participation in a health examination, we found that men who were inactive in their forties had a nearly 40% higher risk of hip fracture compared to their most active counterparts. The protective association between midlife physical activity and hip fracture risk remained statistically significant until the age of 87 years. These findings suggest mid-life physical activity as an important factor for healthy aging, quality of life, and independence into advanced age. This is to the best of our knowledge the first study on hip fracture risk where men have been followed up for an extensive period of time. Combining information on leisure-time physical activity and BMI revealed an even more pronounced association with hip fracture risk. Inactive men with a BMI below 22 kg/m^2^ had a 79% increased risk compared to those who were both active and had a BMI of 25 kg/m^2^ or higher. Among inactive men, there was a trend of increasing hip fracture risk with decreasing BMI, although this was not statistically significant. It has previously been reported that the strongest associations between BMI and hip fracture risk occur at lower BMI levels, reaching a plateau around 25 kg/m^2^, with diminishing benefits at higher levels [27].

Findings similar to ours, on the association between physical activity, BMI, and hip fracture risk, have been reported among postmenopausal women [28]. Our results support long-term benefits of physical activity in midlife and an associated reduction in lifetime risk of hip fracture in men. However, the observed effect may well be attributed to consistent participation in physical activity throughout life and not only a result of the leisure-time physical activity habits in mid-life.

Our findings of a reduced hip fracture risk in physically active men may partly be explained by the mechanical loading on the skeleton posed by weight-bearing exercise, which contributes to enhanced bone health and improved bone mineral density [29]. However, this relationship has been studied less in men than in women. Physical activity improves balance, muscle strength, and coordination, all of which reduce the risk of falling, a critical factor in causing a hip fracture. Despite most bone formation occurring at young age, our findings corroborate the importance of maintaining physical activity in adulthood, which can contribute to maintaining healthy bone structure and attenuating age-related bone loss. There are several possible mechanisms that might account for a higher hip fracture risk among inactive persons. First, a potential change in body composition in inactive persons where inactivity promotes increased fat mass, poor mobility, and possible insulin resistance [30–32]. These metabolic changes may impair bone quality and increase falls risk [33, 34]. Second, being inactive is linked to increased levels of tumor necrosis factor-alpha (TNF-α) and several interleukins, which are associated with chronic low-grade inflammation and ultimately impact both osteoclast and osteoblast activity negatively [35]. Third, inactivity is indirectly associated with a higher degree of comorbidity and greater medication use. Numerous drugs increase the risk of falls, a critical factor in the chain of events that leads to a hip fracture [36].

The observed inverse relationship between BMI and hip fracture risk [15, 16] is thought to be mediated through several mechanisms. First, an increased load on the skeleton from a higher weight enhances bone strength [37], like the mechanical loading from physical activity. Second, the protective padding effect from fat mass around the hips in individuals with a higher BMI reduces the force on the femur in case of a fall [38]. Third, the release of estrogen from adipose tissue, through conversion of testosterone to estrogen by aromatase [39], has a protective effect on bone loss. Emerging evidence suggests that estrogen plays an important role in bone health also in men [40]. Finally, men with a lower BMI may have reduced energy and protein intake compared to men with a higher BMI [41]. Higher energy and protein intake is associated with increased muscle mass and bone mineral density, which contribute to a lower risk of falls and improved bone strength.

### Strengths and limitations

The major strengths of this study include the large study population of men and 1542 hip fracture cases identified during follow-up. The NORHip database provides a virtually complete follow-up of hospital treated hip fractures since 1994. With data available up to 47 years after inclusion, we have been able to follow most participants to either a hip fracture event or death, effectively exhausting the dataset for endpoints. Our follow-up data was available from 1994, at which time the study participants were aged 62–71 years. Any hip fracture occurring earlier than 1994 will not have been captured, underestimating the true hip fracture incidence. However, it is among octogenarians that hip fractures are most common and have the greatest impact on physical health and quality of life. The NORHip database does not distinguish between low and high energy fractures. However, in older people, low bone mineral density appears to be similarly associated with both high- and low-energy fractures [42] and the majority or hip fractures in older people are caused by low energy fractures [43]. Another limitation of this study is its generalizability to today’s population, as the BMI distributions have changed markedly since the 1970s. In population-based studies, information on non-responders is rarely available, and individuals with poor health are commonly underrepresented, potentially introducing selection bias. Residual confounding from unmeasured variables is another limitation and prevents inferences regarding causality. In this study, we have information on leisure time physical activity level at baseline only, and it is a limitation that the study does not assess subsequent changes in physical activity or additional factors that might affect bone, muscles, and fall risks occurring after the initial assessment. Using questionnaires to measure physical activity introduces potential misclassification and self-reporting bias, as responses may be influenced by perceived social desirability [44]. However, previous studies have shown that physical activity tends to be overreported among individuals engaging in low and moderate levels of physical activity, which would attenuate rather than enhance the association between physical activity and hip fracture risk [45]. Ideally, a randomized controlled trial would be the ultimate study design to examine the impact of physical activity and BMI on long-term hip fracture risk. However, the cost, extensive follow-up time, high risk attrition, and implementation challenges make such a study nearly impossible to conduct. Rather, we rely on observational designs, which, despite their limitations, provide valuable information of associations and suggest beneficial effects of physical activity. Our results may have clinical implications, both for men in the same geographical region as the present study and other parts of the world. For men who are middle aged today, it is important that information on the primary preventative measures of physical activity is readily available. The middle-aged years are a window of opportunity for prevention and have the potential to enhance general health decades later. Lifestyle changes in mid-life could alter long-term fracture trajectories. In contrast, older men today who were inactive in middle age would benefit from an earlier and more comprehensive fracture risk assessment and greater focus or treatment with osteoporotic medication after a first fracture. Among men, osteoporosis has historically been underdiagnosed and undertreated representing substantial opportunities for improvement and fracture prevention.

## Conclusion

This study aimed to examine the interplay between physical activity and BMI in 12,900 men aged 40**–**49 years, and their subsequent long-term risk of hip fracture. We found that both factors were independently and jointly associated with long-term hip fracture risk. The protective association of physical activity with hip fracture remained significant until age of 87 years. Men who did not engage in physical activity and had a BMI of under 22 kg/m^2^ had the greatest risk of sustaining a hip fracture. These findings indicate that both maintaining an active lifestyle and a healthy body composition are important for reducing fracture risk later in life. Future research should investigate the mechanisms underlying this long-term protection.

## Data Availability

Due to protection of privacy under the General Data Protection Regulation and Norwegian law, the individual-level data can only be made available after approval by the Regional Committee for Medical and Health Research Ethics and application to the respective data owners (Statistics Norway and the Norwegian Institute of Public Health).

## Declarations

### Disclaimer

Data from the Norwegian Patient Registry have been used in this publication. The interpretation and reporting of these data are the sole responsibility of the authors, and no endorsement by the Norwegian Directorate of Health is intended nor should be inferred.
